# Acute Ischemic Stroke Secondary to Severe Anemia From Upper Gastrointestinal Bleeding: A Case Report

**DOI:** 10.7759/cureus.83820

**Published:** 2025-05-09

**Authors:** Usamah Al-Anbagi, Abdulrahman Saad, Tarek Ibrahim, Abdulqadir J Nashwan

**Affiliations:** 1 Internal Medicine, Hazm Mebaireek General Hospital/Hamad Medical Corporation, Doha, QAT; 2 Medicine, Ministry of Public Health, Doha, QAT; 3 Pharmacy, Hamad Medical Corporation, Doha, QAT; 4 Nursing and Midwifery Research, Hamad Medical Corporation, Doha, QAT

**Keywords:** anemia, computed tomography scan (ct scan), ischemic stroke, magnetic resonance imaging (mri), nonsteroidal anti-inflammatory drug (nsaid), upper gastrointestinal bleeding

## Abstract

Anemia is increasingly recognized as a significant risk factor for ischemic stroke, contributing to cerebral hypoperfusion and exacerbating cerebrovascular events. This case report highlights the complex interplay between severe anemia and ischemic stroke in a 63-year-old male with a history of well-controlled hypertension and type 2 diabetes mellitus. The patient presented with acute neurological deficits, including slurred speech and left-sided weakness, which were later diagnosed as ischemic stroke. His condition was preceded by upper gastrointestinal bleeding from a pyloric channel ulcer, resulting in severe acute posthemorrhagic anemia and consequent hypovolemia. Anemia, often overlooked as a major stroke risk factor, was identified as a key contributor to the ischemic event. Potential mechanisms include reduced oxygen-carrying capacity, cerebral hypoperfusion due to volume loss, and endothelial dysfunction. Immediate management included blood transfusions to correct the anemia, intravenous proton pump inhibitors for gastrointestinal bleeding control, and clopidogrel for secondary stroke prevention. The patient’s condition improved gradually, underscoring the importance of early recognition and treatment of anemia in stroke patients. This case emphasizes the need for comprehensive management of underlying anemia and cerebrovascular risk factors to optimize patient outcomes.

## Introduction

Anemia is characterized by reduced red blood cells (RBCs) or hemoglobin concentration, leading to insufficient oxygen delivery to tissues and organs [1]. It is a common global health problem affecting individuals of all ages and backgrounds [1]. While anemia is frequently associated with systemic symptoms such as fatigue and pallor, its role as a risk factor for cerebrovascular events, particularly ischemic stroke, is often underappreciated [1]. Stroke, the second leading cause of death worldwide, typically results from arterial blockage or insufficient blood flow to the brain [2]. In individuals with anemia, the reduced oxygen-carrying capacity of the blood can exacerbate cerebral hypoperfusion, increasing the risk of ischemic events [3]. This case report discusses a 63-year-old male with a history of well-controlled hypertension and type 2 diabetes mellitus, who initially presented with melena for two days, followed by acute ischemic stroke on the third day, secondary to severe anemia caused by gastrointestinal bleeding. The interplay between anemia and ischemic stroke, as well as the challenges in managing these coexisting conditions, is explored in this report.

## Case presentation

A 63-year-old gentleman with a medical history of hypertension and type 2 diabetes mellitus (diagnosed two years prior, with an HbA1c of 6.5% at diagnosis) presented to the emergency department (ED). His medications included amlodipine 5 mg daily and metformin 500 mg twice daily, with both blood pressure and blood glucose well controlled. He also had a history of peptic ulcer disease, which had been stable with no prior episodes of gastrointestinal bleeding. The patient initially presented to the ED three days before the acute event with complaints of back pain and was discharged on NSAIDs for pain management. Two days later, he returned to the ED with an 8-hour history of slurred speech and left-sided weakness that had developed suddenly. He denied any loss of consciousness, seizures, or sensory deficits.

The patient presented with an 8-hour history of a sudden onset of slurred speech and weakness affecting the left side of the body, involving both the upper and lower limbs. There was no associated facial droop, visual disturbances, dizziness, vertigo, or difficulty swallowing. He denied any sensory deficits, numbness, tingling, or loss of coordination. There were no episodes of loss of consciousness, seizures, headaches, or neck stiffness. No prior similar neurological events were reported, and there was no history of trauma. Neurological examination confirmed slurred speech, reduced grip strength in the left hand, and left-sided weakness (power 4/5 in both the upper and lower limbs). Cranial nerve examination (other than the noted mild dysarthria) was normal, with no facial asymmetry or visual field deficits. Sensory examination was intact to light touch, pinprick, and proprioception bilaterally. Reflexes were symmetrical and within normal limits; plantar responses were downgoing bilaterally. Coordination tests (finger-nose and heel-shin) and cerebellar function were unremarkable. Gait could not be fully assessed due to the acute presentation. The National Institutes of Health Stroke Scale (NIHSS) score was 3. A per rectal examination confirmed the presence of melena. Laboratory investigations revealed severe anemia with a normal mean corpuscular volume (MCV) and mean corpuscular hemoglobin (MCH), and normal iron studies suggested acute blood loss rather than chronic bleeding. All other routine laboratory tests were unremarkable (Table 1).

**Table 1:** Laboratory Investigations ALT: Alanine Aminotransferase; APTT: Activated Partial Thromboplastin Time; AST: Aspartate Aminotransferase; Fe%: Iron Saturation Percentage; g/dL: Grams per Deciliter; g/L: Grams per Liter; INR: International Normalized Ratio; IU/L: International Units per Liter; K: Potassium; MCV: Mean Corpuscular Volume; MCH: Mean Corpuscular Hemoglobin; pg: Picograms; PT: Prothrombin Time; TIBC: Total Iron Binding Capacity; U/L: Units per Liter; μL: Microliter; μmol/L: Micromoles per Liter; LDL: Low-Density Lipoprotein; HDL: High-Density Lipoprotein; RBG: Random Blood Glucose; HbA1c: Hemoglobin A1c.

Parameters	On admission	On discharge	Reference values
Total leukocytes	7.7	4.7	(6.2 x10^3/uL)
Hematocrit	21.8	28.3	(40-50%)
Hemoglobin (gm/dL)	7.2	9.5	(13-17 gm/dL)
Mean corpuscular volume (MCV) (fL)	95.6	90/7	(83-101 fL)
Mean corpuscular hemoglobin (MCH) (pg)	31.6	30.4	(27-32 pg)
Platelet (x10^3/uL)	292	264	(150-410 x10^3/uL)
Iron (umol/L)	23	-	(6-35 umol/L)
Total Iron binding capacity (umol/L)	83	-	(45-80 umol/L)
Transferrin (gm/L)	3.3	-	(2-3.6 gm/L)
Fe% saturation	28	-	(15-45 %)
Serum urea (mmol/L)	3	2.7	(2.5-7.8)
Serum creatinine (umol/L)	67	70	(62-106)
Serum potassium K (mmol/L)	4.2	4	(3.5-5.3)
Serum sodium (mmol/L)	137	139	(133-146)
Serum calcium (mmol/L)	2.33	-	(2.2-2.6)
Serum total protein (gm/L)	64	64	(60-80)
Serum albumin (gm/L)	36	34	(35-50)
ALT (IU/L)	13	12	(0-41)
AST (IU/L)	16	17	(0-41)
Alkaline phosphatase (U/L)	54	50	(40–129)
Serum total bilirubin (mg/dl)	3	8	(0-21)
PT (seconds)	9.7	-	(9.4-12.5 seconds)
INR	0.9	-	<1
APTT (seconds)	30.6	-	(25.1- 36.5 seconds)
Cholesterol	4.2	-	<5.2 mmol/L
Triglyceride	1.1	-	<1.7 mmol/L
LDL	2.2	-	<2.59 mmol/L
HDL	1.4	-	>1 mmol/L
RBG	8.7	-	3.3-5.5 mmol/L
HbA1c	6.8	-	4.8-5.9%

**Figure 1:**
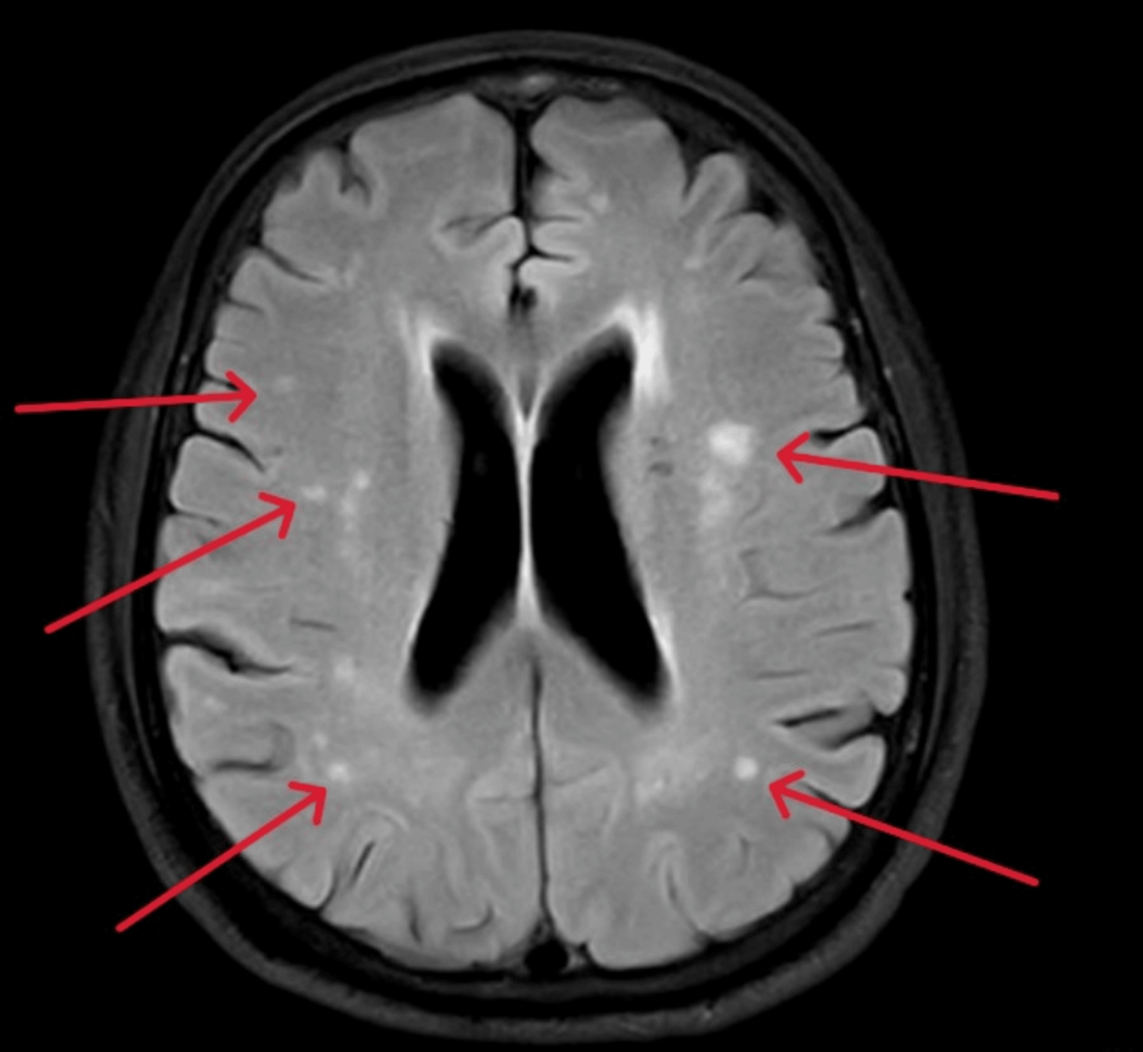
Magnetic Resonance Imaging axial view (Brain) The brain MRI revealed multifocal patchy hyperintense areas and foci, consistent with a recent ischemic infarct (red arrows)

Imaging studies included a head CT scan that showed non-specific changes in the left pericallosal region and basal ganglia. Subsequent MRI of the brain revealed multifocal bilateral cerebral non-hemorrhagic infarcts (Figure 1). An echocardiogram revealed a normal ejection fraction of 57% with mild mitral regurgitation. The patient was referred for cardiology follow-up in the clinic. A carotid Doppler was not done during admission, but was ordered to be completed as an outpatient.

**Figure 2:**
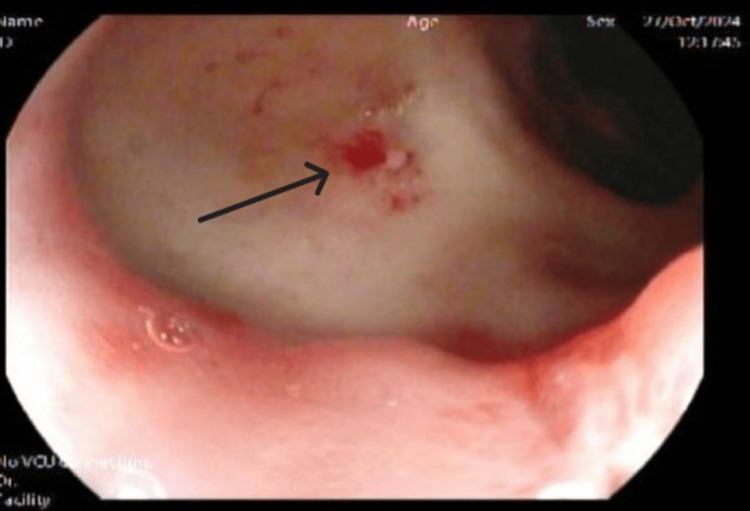
Esophagogastroduodenoscopy (EGD) revealed a pyloric channel ulcer Esophagogastroduodenoscopy (EGD) revealed a pyloric channel ulcer with signs of recent bleeding (black arrow)

**Figure 3:**
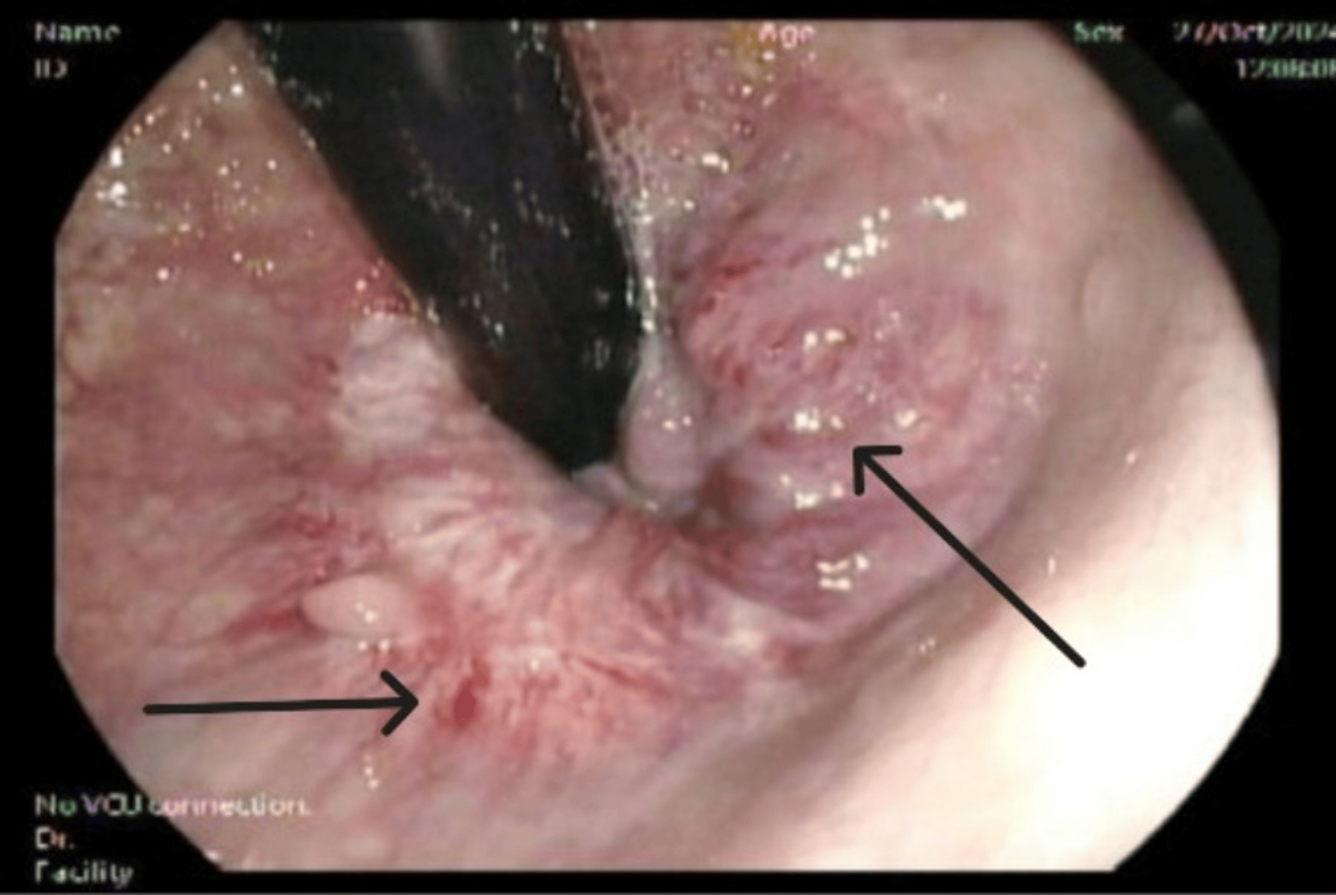
Colonoscopy revealed large congested internal hemorrhoids Colonoscopy, revealed large congested internal hemorrhoids (black arrows)

The patient was admitted as a case of severe anemia due to upper gastrointestinal bleeding complicated by acute ischemic stroke, likely secondary to cerebral hypoperfusion from anemia. He underwent upper esophagogastroduodenoscopy (EGD), which identified a pyloric channel ulcer (Figure 2), and colonoscopy, which revealed large, congested internal hemorrhoids (Figure 3).

During hospitalization, the patient received three units of packed red blood cells to correct the severe anemia and improve cerebral perfusion, with serial monitoring of CBC. Intravenous proton pump inhibitor (PPI) with continuous infusion was initiated to manage the pyloric channel ulcer and prevent further bleeding. Clopidogrel 75 mg and atorvastatin 40 mg were initiated for secondary stroke prevention after agreement with the neurologist and gastroenterologist, with careful consideration of the risk of re-bleeding. The patient was educated on the potential side effects and the importance of adherence.

The patient showed significant improvement during hospitalization and was discharged after 4 days with mild residual left-sided weakness. Discharge medications included clopidogrel 75 mg for secondary stroke prevention, amlodipine 5 mg daily for hypertension, atorvastatin 40 mg, and metformin 500 mg twice daily for diabetes management. Follow-up appointments were scheduled with a physical therapist, gastroenterologist, and neurologist to address the rehabilitation of left-sided weakness, monitor hemoglobin levels, and ensure ongoing management.

## Discussion

This case highlights the complex interplay between anemia and ischemic stroke. A 63-year-old male with a history of well-controlled hypertension, type 2 diabetes mellitus, and severe anemia secondary to recent upper gastrointestinal bleeding (likely due to a gastric ulcer) presented with acute neurological deficits, including slurred speech and left-sided weakness, which were later diagnosed as ischemic stroke. His condition was precipitated by severe anemia, which likely contributed to the ischemic event through cerebral hypoperfusion. While stroke is commonly associated with traditional risk factors such as hypertension, diabetes, and hyperlipidemia, anemia is often overlooked as a significant risk factor that can independently contribute to ischemic events by compromising cerebral perfusion.

Anemia is a condition marked by a reduction in one or more critical RBC parameters, such as hemoglobin concentration, hematocrit, or RBC count, which are typically evaluated through a complete blood count [1]. Anemia, a common condition with a significant global health burden, has gained increasing attention in the context of ischemic stroke, particularly due to its contribution to cerebrovascular events. Anemia is a significant global health challenge affecting individuals in developed and developing countries. According to the World Health Organization (WHO), anemia is defined by hemoglobin levels below 12.0 g/dL for women and 13.0 g/dL for men [1]. In the context of stroke, two main types are recognized: hemorrhagic stroke, characterized by excessive blood within the brain, and ischemic stroke, which results from inadequate blood flow, leading to insufficient oxygen and nutrient supply to brain tissue [2].

Anemia is increasingly acknowledged as a key contributor to cerebrovascular events due to its adverse effects on blood supply and oxygen delivery to the central nervous system [3]. Its impact on morbidity, hospitalization, and mortality is comparable to well-established cardiovascular risk factors like smoking, diabetes, hypertension, and hypercholesterolemia [4-6], earning it the classification of the "fifth cardiovascular risk factor" [7]. Stroke, the second leading cause of death in the U.S., primarily affects individuals over 65, with ischemic strokes being the most common [8].

Anemia is often considered a hyperkinetic state that disrupts endothelial function, leading to a prothrombotic state with potential thrombus formation. Recent studies have explored how the reduced oxygen-carrying capacity in anemia contributes to endothelial damage and thrombus formation [9]. Stroke typically occurs due to brain hypoperfusion from arterial blockage or systemic circulatory failure, where the brain fails to receive enough blood to meet its needs. Two hypotheses explain how anemia can contribute to cerebrovascular events: one suggests that low circulating oxygen triggers increased cerebral blood flow, leading to endothelial damage and thrombosis [10], while the other proposes that anemic hypoxia, similar to systemic hypoperfusion, causes under perfusion in critical brain areas, leaving them vulnerable to ischemic injury [11].

A study using data from the National Health Insurance Service cohort of South Korea analyzed the association between anemia and ischemic stroke in adults diagnosed between 2005 and 2018. The study included 58,699 patients with anemia and a matched control group twice as large. The incidence of ischemic stroke within one year was 0.550% in the anemia group compared to 0.272% in controls, with an adjusted odds ratio (OR) of 1.602 (95% CI, 1.363-1.883). The two-year risk remained elevated, particularly for patients under 50 (OR 2.404, 95% CI, 1.232-4.689; P = .010). Among 623 ischemic stroke patients, anemia was linked to increased mortality risk (HR 1.509, 95% CI, 1.197-1.902; P = .0005). The study confirmed anemia as a risk factor for ischemic stroke and post-stroke mortality, emphasizing the importance of anemia management in stroke patients [12].

Clinical assessment plays a crucial role in the early diagnosis of ischemic stroke, starting with the collection of medical history, including past conditions such as hypertension, diabetes, cardiovascular diseases, and any previous strokes or transient ischemic attacks. The physical examination focuses on vital signs, such as blood pressure, heart rate, temperature, and oxygen saturation, as sudden changes, like elevated BP or irregular heart rhythms, may suggest an acute ischemic event. A detailed neurological exam evaluates motor, speech, and sensory functions to help determine the stroke's location and severity. Non-contrast CT scans are typically the first line of imaging in suspected ischemic stroke, but they may not detect small or subtle infarcts, especially in the hyperacute phase. If clinical suspicion remains high despite a negative CT scan, advanced imaging techniques such as magnetic resonance angiography (MRA) or diffusion-weighted imaging (DWI) can offer higher sensitivity [13,14].

The management of this patient involved a multi-faceted approach addressing both the ischemic stroke and the underlying anemia. Immediate treatment focused on stabilizing the patient’s anemia with blood transfusions. This is crucial for improving oxygen delivery to cerebral tissues, correcting the hypoxic state that likely contributed to the ischemic stroke, and preventing further neurological damage [15,16]. In addition to blood transfusions, the patient was treated with PPI to control ongoing gastrointestinal bleeding and prevent further episodes [17]. Given the ischemic nature of the stroke, antiplatelet therapy with clopidogrel was initiated to avoid thromboembolic complications, as aspirin carries a higher risk of bleeding [18]. This case emphasizes the importance of early recognition and management of anemia in stroke patients, as well as the necessity for prompt correction of underlying bleeding sources. While the patient’s outcome was positive, with gradual improvement in neurological function, it is essential to continue long-term monitoring for any potential recurrence of gastrointestinal bleeding, stroke, or anemia to prevent further complications.

## Conclusions

This case underscores the importance of considering anemia as a significant and potentially modifiable risk factor in patients presenting with ischemic stroke. The patient’s clinical course highlights the crucial need for early recognition and treatment of anemia, particularly in the setting of comorbidities such as hypertension and diabetes. Prompt management of anemia with blood transfusions, treatment for the underlying cause of bleeding, and stroke prevention can improve patient outcomes. Ultimately, this case exemplifies the necessity of a comprehensive approach to managing both ischemic stroke and anemia, emphasizing the value of addressing all contributing factors to reduce morbidity and mortality in stroke patients.
